# Protective Effects of Necrostatin-1 in Acute Pancreatitis: Partial Involvement of Receptor Interacting Protein Kinase 1

**DOI:** 10.3390/cells10051035

**Published:** 2021-04-27

**Authors:** Yulin Ouyang, Li Wen, Jane A. Armstrong, Michael Chvanov, Diane Latawiec, Wenhao Cai, Mohammad Awais, Rajarshi Mukherjee, Wei Huang, Peter J. Gough, John Bertin, Alexei V. Tepikin, Robert Sutton, David N. Criddle

**Affiliations:** 1 Department of Molecular Physiology & Cell Signalling, Institute of Systems, Molecular & Integrative Biology, University of Liverpool, Liverpool L69 3BX, UK; yl.ouyang@siat.ac.cn (Y.O.); chvanov@liverpool.ac.uk (M.C.); kiev@liverpool.ac.uk (A.V.T.); 2 Brain Cognition and Brain Disease Institute, Chinese Academy of Sciences, Shenzhen 518055, China; 3 Molecular & Clinical Cancer Medicine, Institute of Systems, Molecular & Integrative Biology, University of Liverpool, Liverpool L69 3BX, UK; li.wen@shgh.cn (L.W.); janearm@liverpool.ac.uk (J.A.A.); latawiec@liverpool.ac.uk (D.L.); Wenhao.Cai@liverpool.ac.uk (W.C.); awais@liverpool.ac.uk (M.A.); rishim@liverpool.ac.uk (R.M.); wei.huang.scu@vip.163.com (W.H.); sutton@liverpool.ac.uk (R.S.); 4 Pattern Recognition Receptor Discovery Performance Unit, Immuno-Inflammation Therapeutic Area, GlaxoSmithKline, Collegeville, PA 19426, USA; Pgough@inzentx.com (P.J.G.); John.Bertin@sanofi.com (J.B.)

**Keywords:** acute pancreatitis, receptor-interacting protein kinase 1, RIPK1, necrostatin-1, necroptosis, cell death, indoleamine 2,3-dioxygenase, epacadostat

## Abstract

Acute pancreatitis (AP) is a severe and potentially fatal disease caused predominantly by alcohol excess and gallstones, which lacks a specific therapy. The role of Receptor-Interacting Protein Kinase 1 (RIPK1), a key component of programmed necrosis (Necroptosis), is unclear in AP. We assessed the effects of RIPK1 inhibitor Necrostatin-1 (Nec-1) and RIPK1 modification (RIPK1^K45A^: kinase dead) in bile acid (TLCS-AP), alcoholic (FAEE-AP) and caerulein hyperstimulation (CER-AP) mouse models. Involvement of collateral Nec-1 target indoleamine 2,3-dioxygenase (IDO) was probed with the inhibitor Epacadostat (EPA). Effects of Nec-1 and RIPK1^K45A^ were also compared on pancreatic acinar cell (PAC) fate in vitro and underlying mechanisms explored. Nec-1 markedly ameliorated histological and biochemical changes in all models. However, these were only partially reduced or unchanged in RIPK1^K45A^ mice. Inhibition of IDO with EPA was protective in TLCS-AP. Both Nec-1 and RIPK1^K45A^ modification inhibited TLCS- and FAEE-induced PAC necrosis in vitro. Nec-1 did not affect TLCS-induced Ca^2+^ entry in PACs, however, it inhibited an associated ROS elevation. The results demonstrate protective actions of Nec-1 in multiple models. However, RIPK1-dependent necroptosis only partially contributed to beneficial effects, and actions on targets such as IDO are likely to be important.

## 1. Introduction

Acute pancreatitis (AP) is a painful, debilitating inflammatory disease with significant mortality. The incidence of AP has increased in recent decades and places a considerable burden on health-care provision [1]. Core to the development of AP is damage to exocrine tissue, with extensive parenchymal necrosis that determines clinical outcome; in severe cases a systemic inflammatory response syndrome (SIRS), multiple organ failure and patient death may ensue. However, our understanding of cell death mechanisms in AP is incomplete and currently there is no specific therapy for the disease; identification and development of novel approaches is therefore paramount [2].

Studies have demonstrated that a variety of AP precipitants including bile acids, non-oxidative ethanol metabolites (fatty acid ethyl esters: FAEEs) and cholecystokinin hyperstimulation, raise cytosolic Ca^2+^ in the pancreatic acinar cell (PAC) in a sustained manner, causing opening of the mitochondrial permeability transition pore (MPTP), mitochondrial depolarization, rundown of ATP production and necrosis [3,4,5]. Necrosis is considered a largely uncontrolled event that results in lysis of the cell membrane, allowing escape of cellular contents into the interstitial compartment. Necroptosis, a programmed form of necrosis, has the same endpoint as necrosis but is differentiated by integral activation of receptor interacting protein kinase 1 (RIPK1), RIPK3 and mixed lineage kinase domain-like protein (MLKL). It constitutes a regulated cell death programme in response to activation of death receptors, Toll- and NOD-like receptors, T cell receptors, genotoxic stress and viruses [6]. RIPK1 involvement has been shown in necroptosis-associated disease, including myocardial infarction, stroke, neurodegeneration and ischaemia-reperfusion injury [7,8,9].

However, relatively few studies have addressed the involvement of necroptosis in AP; so far these have produced mixed results, with many relying on a single experimental model. Genetic knockout studies have shown that RIPK3^−/−^ mice were protected from caerulein (CER)-AP and taurolithocholic acid sulphate (TLCS)-AP [10,11,12], while MLKL^-/-^ ameliorated CER-AP [13]. In contrast, RIPK1^KD/KD D138N^ and RIPK1 P-loop deficient RIP1^Δ/Δ^ mice were not protected from CER-AP [12,14]. Similarly, there have been differing outcomes in studies assessing pharmacological inhibition of necroptosis. For example, the RIPK1 inhibitor necrostatin-1 (Nec-1) was unprotective in CER-AP, and moreover increased pancreatic damage when combined with the pan-caspase inhibitor zVAD [15]. Conversely, protective effects of Nec-1 have been reported in CER-AP, TLCS-AP [10] and L-arginine-induced pancreatitis [16], although to-date no studies have examined the effects of Nec-1 and the role of necroptosis in alcoholic AP. Interestingly, although Nec-1 has been widely used as an inhibitor of RIPK1, it possesses other targets including indoleamine 2,3-dioxygenase (IDO), an enzyme involved in tryptophan metabolism. Recent seminal work by the Mole group has demonstrated the importance of the kynurenine pathway, downstream of IDO, in the development of AP both in patient and animal studies and is considered a promising therapeutic target [17,18].

Here we have evaluated the involvement of RIPK1 in AP using a RIPK1 kinase-dead (RIPK1^K45A^) mouse and pharmacological inhibition in three in vivo models reflecting the principal aetiologies, including our recent alcoholic AP model (FAEE-AP) [4]. These were combined with respective in vitro cell death assays. The results demonstrate that while Nec-1 treatment markedly ameliorated AP in all models, RIPK1 kinase modification caused only partial or no protection, indicating only a minor role of RIPK1-mediated necroptosis. Pharmacological inhibition of IDO was protective in AP and actions of Nec-1 on collateral targets are likely to be important for its beneficial effects.

## 2. Materials and Methods

### 2.1. Animals

For in vivo and in vitro experiments, 8–10 weeks old male C57BL/6J mice (Charles River Ltd. Margate, Kent, UK) and male RIPK1^K45A^ mice were used. RIPK1^K45A^ mice were generously provided by GlaxoSmithKline [19]. Genotyping of mice was performed using standard PCR with a primer set (69277flp-JEM2, 5′-CTCTGATTGCTTTATAGGACACAGCACTAAGC-3′; 69278flp-JEM2, 5′-GTCTTCAGTGATGTCTTCCTCGTATATTTCTCAAG-3′; 473bp for the Wild-type allele, 575bp for the RIPK1^K45A^ allele).

### 2.2. Experimental Acute Pancreatitis

TLCS-AP was induced by pancreatic ductal infusion of 3 mM TLCS at 5 μL/min for 10 min via an infusion pump as described previously [20]; FAEE-AP was induced by 2 hourly intraperitoneal injections of 1.35 g/kg ethanol and 150 mg/kg palmitoleic acid (POA) [4], and mice sacrificed 24 h later. CER-AP was induced by 7 hourly intraperitoneal cerulein injections at a dose of 50 mg/kg [3] and mice sacrificed 12 h later. In all models, analgesia was administered using 0.1 mg/kg buprenorphine hydrochloride (Temgesic, Reckitt and Coleman, Hull, UK). All experimental protocols were approved by the local ethics committee (University of Liverpool). Nec-1 (Sigma-Aldrich, N9073, Dorset, UK) was dissolved in 10% DMSO + 90% PEG 400 and administered at a dose of 56 mg/kg consistently for 12/24 h via subcutaneous osmotic mini-pump (Charles River UK, Ltd., ALZET osmotic mini-pumps (2001D), Cambridge, UK). Epacadostat (EPA) (Selleck Chemicals, INCB24360, Cambridge, UK) was dissolved in 10% DMSO + 90% PEG 400 and delivered through osmotic mini pump at the dose of 50 mg/kg [21]. Osmotic mini-pumps for treatments (Nec-1, EPA) were inserted into the mice 0.5 h after TLCS-AP induction, after the 2nd injection of POA in FAEE-AP and after the 3rd injection of caerulein in CER-AP [22].

### 2.3. Histological Analysis and Biochemical Measurements of Acute Pancreatitis

Pancreatic tissue was collected and fixed in 10% formalin, embedded in paraffin and stained with H and E. Scoring was performed on 10 random fields by 2 blinded investigators independently grading edema, inflammatory cell infiltration, and acinar necrosis (scale; 0–3) as described previously [3] and data presented as the mean ± SEM (≥5 mice/group). Pancreatic trypsin activity was determined with an established protocol [22] using trypsin peptide Boc-Gln-Ala-Arg-MCA substrate (Peptides International, Louisville, KY, USA) with excitation at 380 nm and emission at 440 nm. Serum amylase was determined using a Roche Analyzer; serum interleukin (IL)-6 was determined by enzyme-linked immunosorbent assay (R&D Systems); myeloperoxidase (MPO) activity was measured according to an established protocol [22] using a plate reader, calculated as the difference between 0 and 3 min at an absorbance wavelength of 655 nm. Histological and biochemical responses were normalized to control changes after AP induction in C57BL/6J mice for each model, to compare the effects of Nec-1 treatment or RIPK1^K45A^ modification across AP models.

### 2.4. Cell Preparation and Solutions

Freshly isolated murine PACs were obtained from the pancreas of 8–10-week-old C57BL/6J or RIPK1^K45A^ mice using a standard collagenase digestion procedure [4]. The extracellular solution contained (mM): 140 NaCl, 4.7 KCl, 1.13 MgCl_2_, 1 CaCl_2_, 10 D-glucose, and 10 HEPES. The final pH of the solution was adjusted to pH 7.35. All experiments on isolated PACs were performed no more than 4 h after isolation unless otherwise stated.

### 2.5. Immunofluorescence

Freshly isolated murine PACs were added to poly-L-lysine coated 35 mm glass bottom dishes and left to adhere for 30 min at room temperature (RT). Cells were then fixed in 4% paraformaldehyde for 20 min, permeabilized with 0.2% Triton X-100 for 5 min, blocked with 10% goat serum and 1% bovine serum albumen in PBS for 1 hr at RT (BSA; Sigma-Aldrich, A3294, Dorset, UK), then incubated with primary antibodies against RIPK1 (Mouse Anti-RIP from BD Transduction Lab: BD610458, 1:200, San Diego, CA, USA), RIPK3 (Rabbit Anti-RIP3 from Abcam: ab152130; 1:100, Cambridge, UK) 1 hr at RT then incubated with corresponding secondary antibody(s), Goat anti-mouse (H + L) lgG (Alexa Fluor 488, A-11001 Invitrogen,1:1000, Loughborough, UK) and Goat anti-rabbit (H + L) lgG (Alexa Fluor 647, A-21244 Invitrogen, 1:500, Loughborough, UK), for 30 min at RT in the dark. Cells were preserved in Azide PBS prior to imaging.

### 2.6. Western Blotting

Isolated PACs were unstimulated or treated with TLCS for 0 h, 2 h and 4 h at 37 °C. Protein was extracted by radioimmunoprecipitation assay (RIPA) buffer (Sigma-Aldrich, R0278, Dorset, UK) containing protease inhibitor cocktail (Thermo Scientific, 87785, Loughborough, UK), phosphatase cocktail 2 (Sigma-Aldrich, P5726) and phosphatase cocktail 3 (Sigma-Aldrich, P0044, Dorset, UK) rotated for 30 min and centrifuged at 16,000 *g* for 15 min at 4 °C. Protein concentration was determined by the BCA assay (Thermo Fisher Scientific, Loughborough, UK). Proteins were separated by SDS-PAGE using a NuPAGE^TM^ 4–12% Bis-Tris Protein Gels (ThermoFisher scientific, Loughborough, UK) and transferred onto nitrocellulose membranes. Non-specific binding was blocked by 3% (*w/v*) non-fat milk in PBS for 1 h. Blots were then incubated at 4 °C overnight with primary antibody to cytochrome C (BD Pharmingen^TM^: 556433, 1:500, San Diego, CA, USA), Calnexin (Sigma, C4731, 1:1000, Dorset, UK), RIPK1 (Mouse Anti-RIP from BD Transduction Lab: BD610458, 1:200, San Diego, CA, USA), RIPK3 (Rabbit Anti-RIP3 from Abcam: ab152130; 1:200, Cambridge, UK) in 3% (*w/v*) non-fat milk in PBS. After the first antibody, the membranes were washed once with 0.05%-tween in PBS and 3 times with PBS (5 min each). Then they were incubated for 1 h with corresponding peroxidase-labelled secondary antibody in 3% (*w/v*) non-fat milk in PBS. Blots were developed for visualization using an enhanced chemiluminescence (ECL) detection kit (Thermo Scientific, Loughborough, UK) through a Bio-Rad ChemiDoc^TM^ XRS + System. The pixel intensities of the bands were calculated using ImageLab software.

### 2.7. Measurement of Necrosis and Apoptosis

Confocal imaging was performed using a Zeiss LSM710 system. Freshly isolated murine PACs were stimulated with either TLCS (500 μM) or POAEE (100 μM) at room temperature for 30 min with or without Nec-1 (30 µM). The total cell number was detected by Hoechst 33342 (10 µg/mL; excitation 364 nm, emission 405–450 nm; Molecular Probes, H3570) and necrosis measured by Propidium Iodide (PI) (10 μg/mL; excitation 488 nm, emission 630–693 nm; Sigma, P4170, Waltham, MA, USA). The total number of cells showing PI uptake in each group was counted from at least 12 random fields and presented as a percentage of the total cell number. Freshly isolated murine PACs were incubated with CellEvent^®^ Caspase 3/7 green (Life Technologies, C10423, Waltham, MA, USA)) with or without Nec-1 (30 µM) for 30 min at 37 °C and then stimulated with TLCS (500 μM) or POAEE (100 μM) to induce apoptosis over an 8 h period (excitation filter 540 nM; emission filter 590 nM) in POLARstar Omega Plate Reader (BMG Labtech, Ortenberg, Germany).

### 2.8. Measurement of Cytosolic Calcium and Reactive Oxygen Species

Fluorescence imaging was performed using an Olympus IX71 based inverted imaging system (Till Photonics GmbH, Munich, Germany). Freshly isolated murine PACs were loaded with Fura2-AM (3 μM; excitation at 340 and 380 nm, emission recorded with 510 nm filter) to measure cytosolic calcium ([Ca^2+^]_c_). The cells were attached to cover slips coated with poly-L-lysine (0.01%) and placed in an open chamber on the microscope stage and perfused with extracellular solution (see above) to obtain a stable baseline (180–200 s). For ROS measurements, PACs were loaded with 5 μM chloromethyl-2,7-dichlorodihydrofluorescein diacetate (CM-H_2_DCFDA) with and without Nec-1 for 30 min at 37 °C. Cells were placed into a 96 well plate-reader and ROS production measured in response to stimulation with AP toxins at 37 °C for 5 h (excitation 488 nm and emission 520 nm; POLARstar Omega Plate Reader; BMG Labtech, Ortenberg, Germany).

### 2.9. Statistical Analysis

In order to compare results from the three in vivo AP models together, responses after AP induction in WT were averaged and normalization (to 100) was performed for each model: (intervention group/AP induction in WT) × 100. Prism 5.0 software (GraphPad Software Inc., La Jolla, CA, USA) was used to perform statistical analyses, using ANOVA with Tukey post hoc test or a Student’s *t*-test as appropriate. Results are presented as mean ± SEM obtained from three or more independent experiments. *p* values of < 0.05 were considered to indicate significant differences.

## 3. Results

### 3.1. Effects of Genetic and Pharmacological RIPK1 Inhibition in FAEE-AP, TLCS-AP and CER-AP

The genotyping of RIPK1^K45A^ homozygous mice revealed a distinct band at 575 bp denoting RIPK (Figure S1a). In PACs from RIPK1^K45A^ and WT mice there was a similar expression and distribution of RIPK1 and RIPK3, assessed by Western blotting and confocal immunofluorescence (Figure S1b,c).

**Figure 1:**
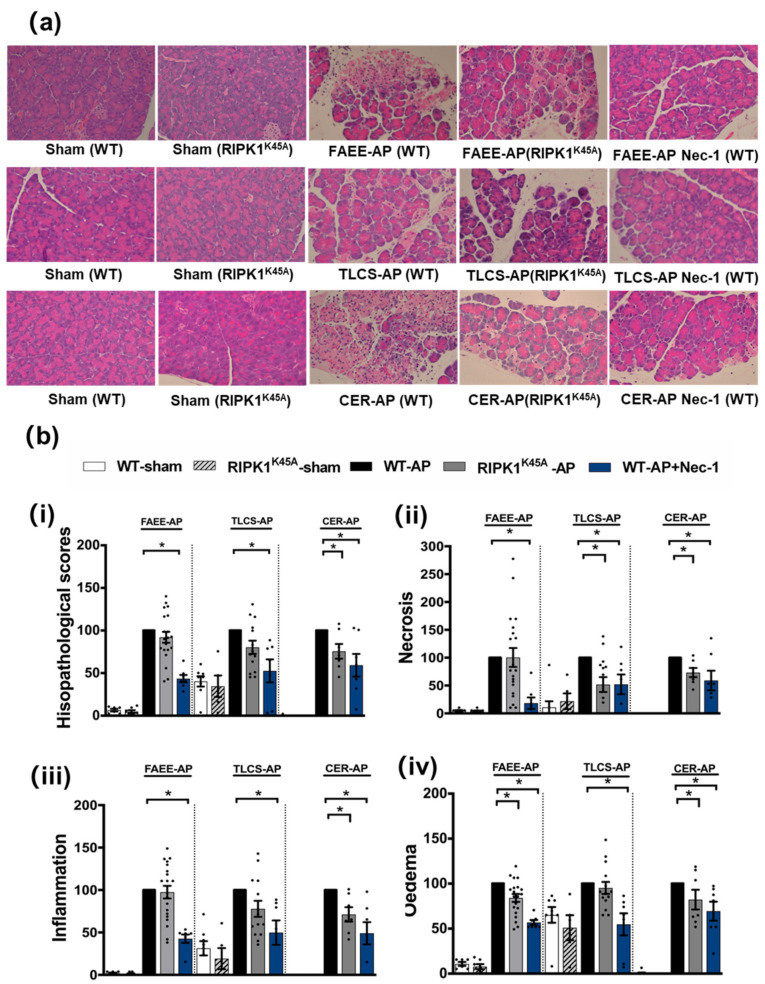
Comparison of histological damage in FAEE-AP, TLCS-AP and CER-AP in RIPK1^K45A^ and WT mice. (**a**) Representative H and E pancreas images (×200) from sham (control), FAEE-AP, TLCS-AP, CER-AP in RIPK1^K45A^, WT, and WT with Nec-1 treatment. (**b**) Pancreatic histology scores ((**i**) total score, (**ii**) necrosis, (**iii**) inflammation and (**iv**) oedema) in three AP models. Each dot represents a mouse. Responses were normalized to control changes after AP induction in WT and are expressed as the mean ± SEM (≥5 mice/group). Significant differences between RIPK1^K45A^ or Nec-1 treatment groups from control are shown as * *p* < 0.05.

**Figure 2:**
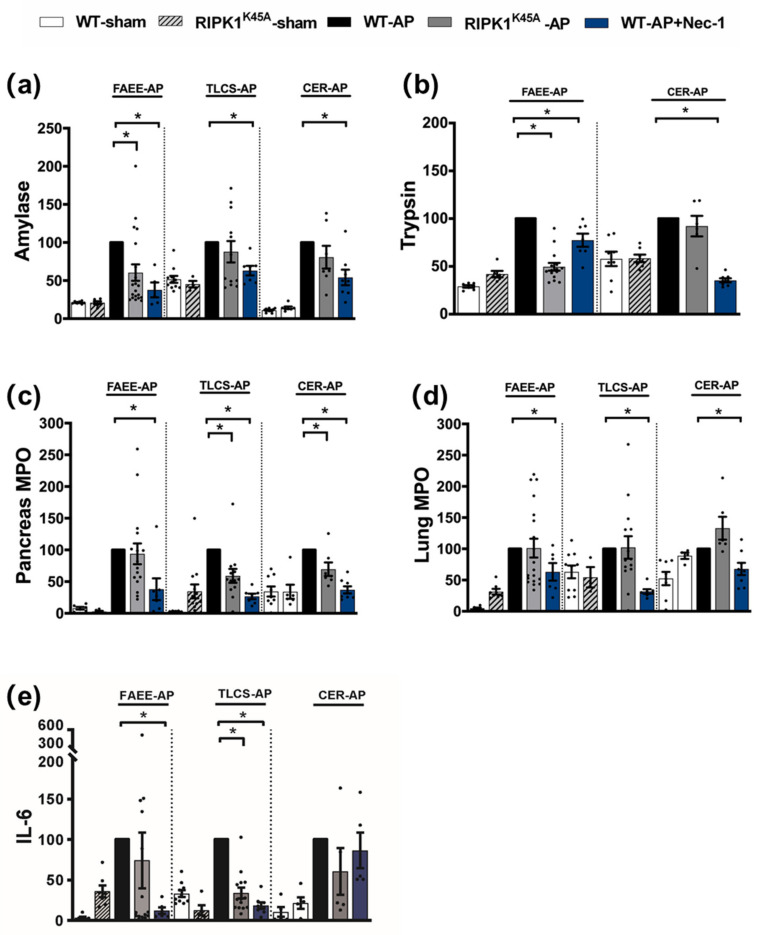
Comparison of biochemical changes in FAEE-AP, TLCS-AP and CER-AP in RIPK1^K45A^ and WT mice. Changes in the levels of (**a**) amylase, (**b**) trypsin, (**c**) pancreatic myeloperoxidase (MPO), (**d**) lung MPO and (**e**) interleukin-6 (IL-6) are shown in three AP models. Each dot represents a mouse. Responses were normalized to control changes after AP induction in WT and are expressed as the mean ± SEM (≥5 mice/group). Significant differences between RIPK1^K45A^ or Nec-1 treatment groups from control are shown as * *p* < 0.05.

Induction of AP in 3 distinct models (TLCS-AP, FAEE-AP and CER-AP) caused significant histopathological pancreatic damage (Figure 1a), consistent with previous studies [4,22]. In RIPK1^K45A^ mice there was no significant reduction of total histopathological scores in either FAEE-AP or TLCS-AP compared to control WT mice, although there was partial protection in CER-AP (Figure 1a,b). In contrast, Nec-1 treatment reduced pancreatic damage in all experimental AP models, with significantly reduced oedema, inflammation and necrosis scores (Figure 1a,b). Similarly, Nec-1 treatment significantly improved biochemical parameters in all 3 AP models (Figure 2). Consistent with histological data, the effects of RIPK1^K45A^ on biochemical parameters did not reflect those of pharmacological inhibition, with either no or partial inhibition observed in the AP models. Thus, whereas Nec-1 reduced amylase elevations in all three models, RIPK1^K45A^ modification caused only a partial reduction in FAEE-AP, with no significant effect in either TLCS-AP or CER-AP (Figure 2a). Both Nec-1 treatment and RIPK1^K45A^ modification decreased elevated trypsin levels in FAEE-AP, however, only Nec-1 treatment was protective in CER-AP (Figure 2b). Raised pancreatic and lung MPO levels were greatly reduced by Nec-1 treatment in all AP models. However, RIPK1^K45A^ only partially decreased the former in TLCS-AP and CER-AP, with no effect in FAEE-AP (Figure 2c) and was without effect on raised lung MPO levels in all models (Figure 2d). Increases of IL-6 in FAEE-AP and TLCS-AP were significantly reduced by Nec-1 treatment, whereas RIPK1^K45A^ was only protective in TLCS-AP (Figure 2e).

### 3.2. Protective Effects of IDO Inhibition in TLCS-AP

**Figure 3:**
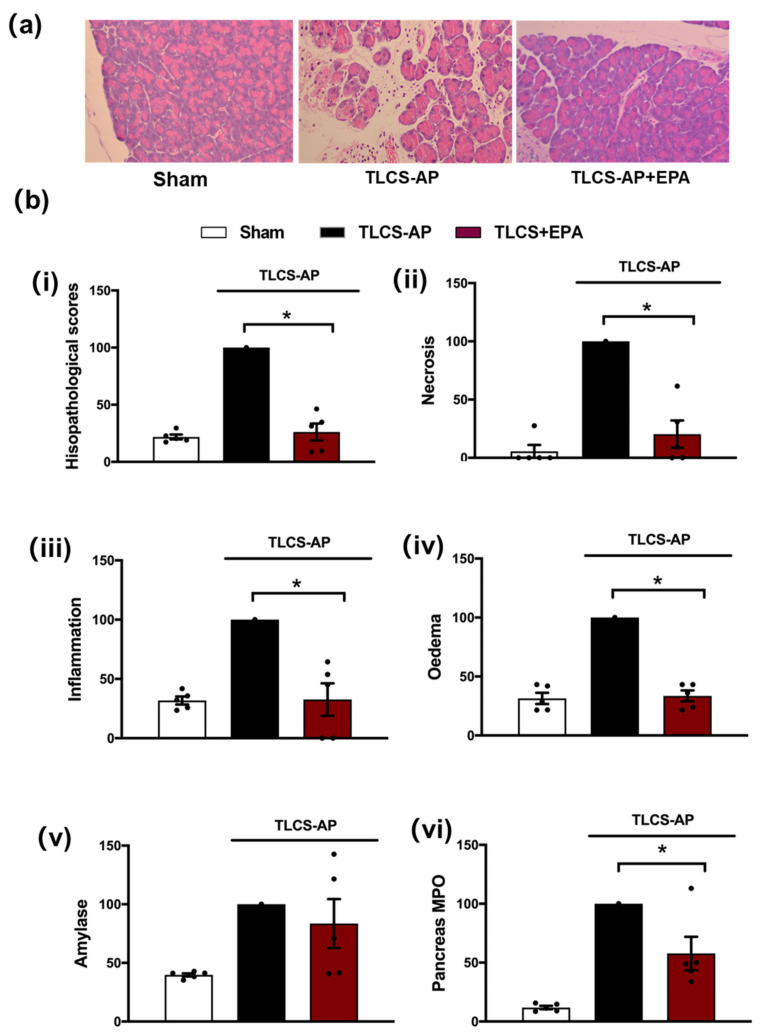
Protective effects of Epacadostat in TLCS-AP. (**a**) Representative H and E pancreas images (×200) from sham (control) and TLCS-AP in WT, with and without epacadostat (EPA) treatment. Changes in (**b**) pancreatic histological damage ((**i**) total score, (**ii**) necrosis, (**iii**) inflammation and (**iv**) oedema and biochemical alterations of (**v**) amylase and (**vi**) pancreas myeloperoxidase (MPO) in TLCS-AP with and without EPA treatment. Each dot represents a mouse. Responses were normalized to control changes after AP induction in WT and are expressed as the mean ± SEM (≥5 mice/group). Significant differences between the EPA treatment group and control are shown as * *p* < 0.05.

A secondary target of Nec-1, IDO, was subsequently investigated using the TLCS-AP model. Inhibition of IDO with Epacadostat (EPA) treatment markedly decreased local pancreatic damage, with significant reductions of oedema, inflammation and necrosis (Figure 3a,b). In addition, EPA reduced the elevated pancreatic MPO in TLCS-AP, with a trend to lower serum amylase in EPA-treated compared to the non-treated group, although this did not attain significance (Figure 3b).

### 3.3. Comparative Effects of RIPK1^K45A^ and Nec-1 on PAC Cell Death

**Figure 4:**
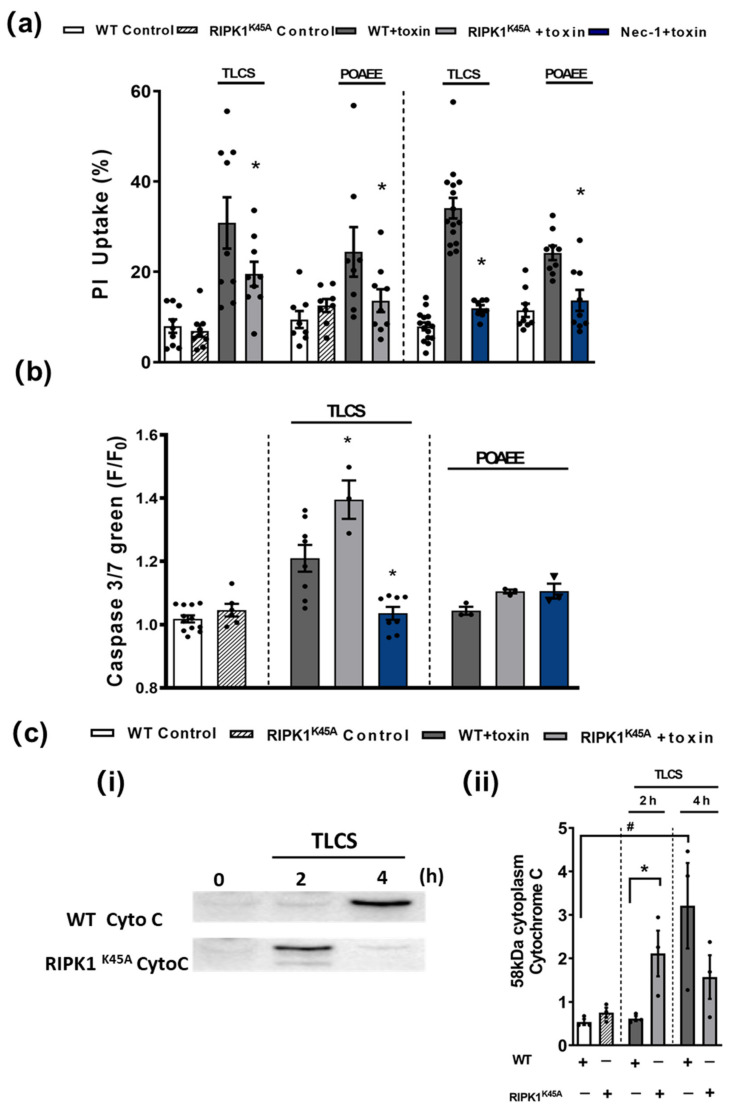
Effects of RIPK1K^45A^ and Nec-1 on TLCS- and POAEE-induced pancreatic acinar cell (PAC) death. (**a**) Bar graph showing the effects of RIPK1^K45A^ modification and Nec-1 (10 μM) on TLCS (500 μM)- and POAEE (100 μM)-induced necrosis (propidium iodide (PI) uptake). Changes are normalized as the ratio of total cell number to necrotic cell number. (**b**) Bar graph showing the effects of RIPK1^K45A^ modification and Nec-1 (10 μM) on TLCS (500 μM) and POAEE (100 μM) induced apoptosis (Caspase-3/7 green). The data are normalized as F/F_0_. (**c**) (**i**) Representative western blot images showing cytochrome C (58 KD) protein level in WT and RIPK1^K45A^ PACs at 0, 2 and 4 h with or without TLCS (500 μM). (**ii**) Bar graph showing the quantification of cytoplasm cytochrome C levels in WT and RIPK1^K45A^ PACs at 0, 2 and 4 h with or without TLCS (500 μM). Each dot represents a mouse. All data are expressed as the mean ± SEM (*n* ≥ 3 mice/group). Significant differences are shown as * *p* < 0.05 and ^#^ *p* < 0.05.

In in vitro cell death assays no differences were detected in the basal levels of necrosis between RIPK1^K45A^ and WT PACs or of apoptosis between RIPK1^K45A^ and WT PACs (Figure 4). Both TLCS and POAEE caused necrosis that was significantly reduced by the RIPK1^K45A^ modification and by Nec-1 application in WT (Figure 4a). TLCS also increased apoptotic cell death in RIPK1^K45A^ and WT PACs, while POAEE was without effect. There was a significantly greater induction of apoptosis in response to TLCS in the RIPK1^K45A^ mice compared to WT. Nec-1 significantly reduced TLCS-induced apoptosis to control levels, whereas it did not alter apoptosis levels in the presence of POAEE (Figure 4b). Furthermore, the appearance of cytosolic cytochrome C in response to TLCS was accelerated in RIPK1^K45A^ PACs compared to WT (Figure 4c), consistent with enhanced apoptosis observed in the cell death assays.

### 3.4. Comparative Effects of RIPK1^K45A^ and Nec-1 on Intracellular Ca^2+^ and ROS

**Figure 5:**
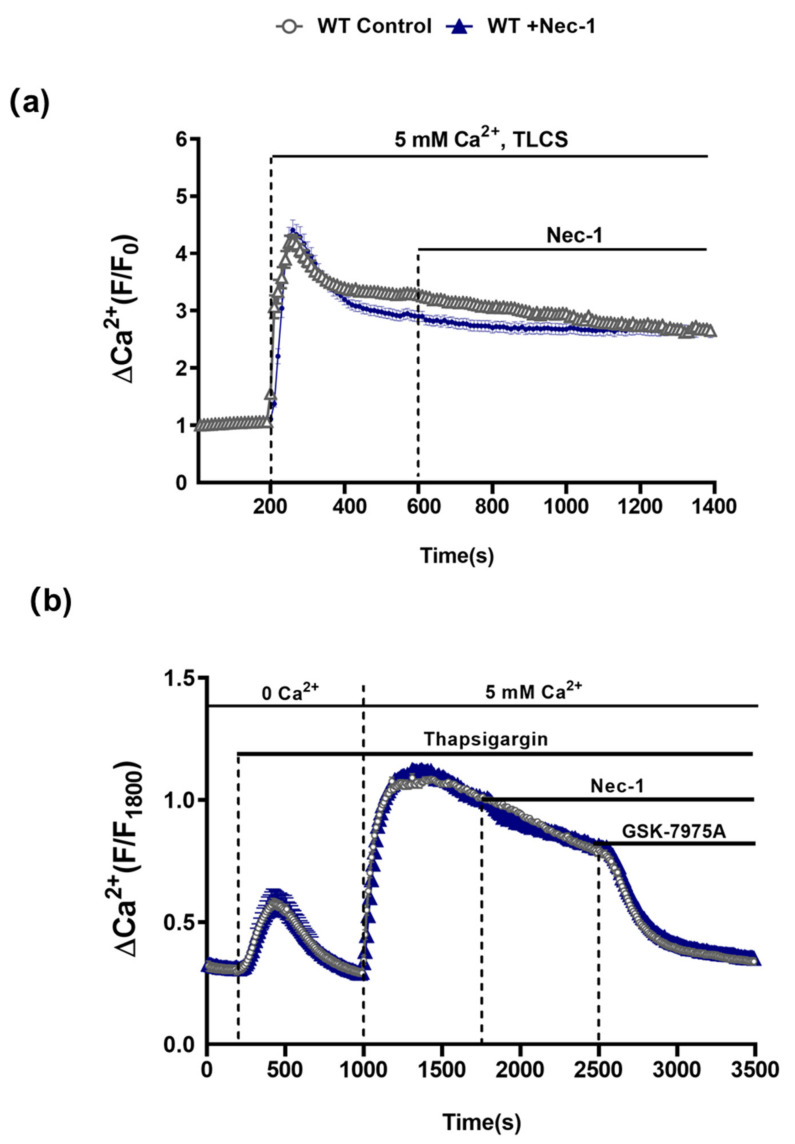
Nec-1 does not inhibit Ca^2+^ entry mechanisms in PACs. (**a**) Graph showing the effects of Nec-1 (30 μM) on TLCS (500 μM)-induced sustained cytosolic Ca^2+^ elevations (Fura2-AM) in PACs. TLCS (500 μM) was applied at 200s (blue; n = 97 cells) and effects of Nec-1, added at 600 s, were compared to time-matched controls (black; n = 77 cells). (**b**) Graph showing the effects of Nec-1 (30 μM) on store-operated Ca^2+^ entry (SOCE) in PACs. Thapsigargin was applied under Ca^2+^-free conditions to elicit internal store depletion. Subsequently 5 mM Ca^2+^ was added from 1000 s to 1800 s to induce SOCE; effects of Nec-1 applied at 1800 s (blue; n = 66 cells) were compared to time-matched controls (black; n = 54 cells). The Orai1 inhibitor GSK-7975A (10 μM) was applied as a positive control at 2500 s to reverse the SOCE. Changes are normalized increases in fluorescence from the baseline (F/F_0_) in (**b**) and (F/F_1800_) in (**c**) and data expressed as the mean ± SEM (*n* ≥ 3 mice/group).

In order to assess the possible involvement of RIPK1 in PAC [Ca^2+^]_c_ overload that leads to necrosis [22,23], the effects of Nec-1 on TLCS-induced [Ca^2+^]_c_ elevations in isolated PACs from WT mice were investigated. Application of TLCS (500 µM) induced a typical rapid peak elevation of [Ca^2+^]_c_ followed by a sustained plateau. However, subsequent addition of Nec-1 did not inhibit the sustained Ca^2+^rise (Figure 5a). Furthermore, in separate experiments Nec-1 did not alter store-operated Ca^2+^ entry induced by depletion of intracellular stores with the SERCA pump inhibitor thapsigargin; the sustained increase of [Ca^2+^]_c_ upon readmission of extracellular Ca^2+^ was unaffected by Nec-1. In contrast, addition of the Orai-1 inhibitor GSK-7975A suppressed the store-operated Ca^2+^ influx (Figure 5b), consistent with previous findings [22].

**Figure 6:**
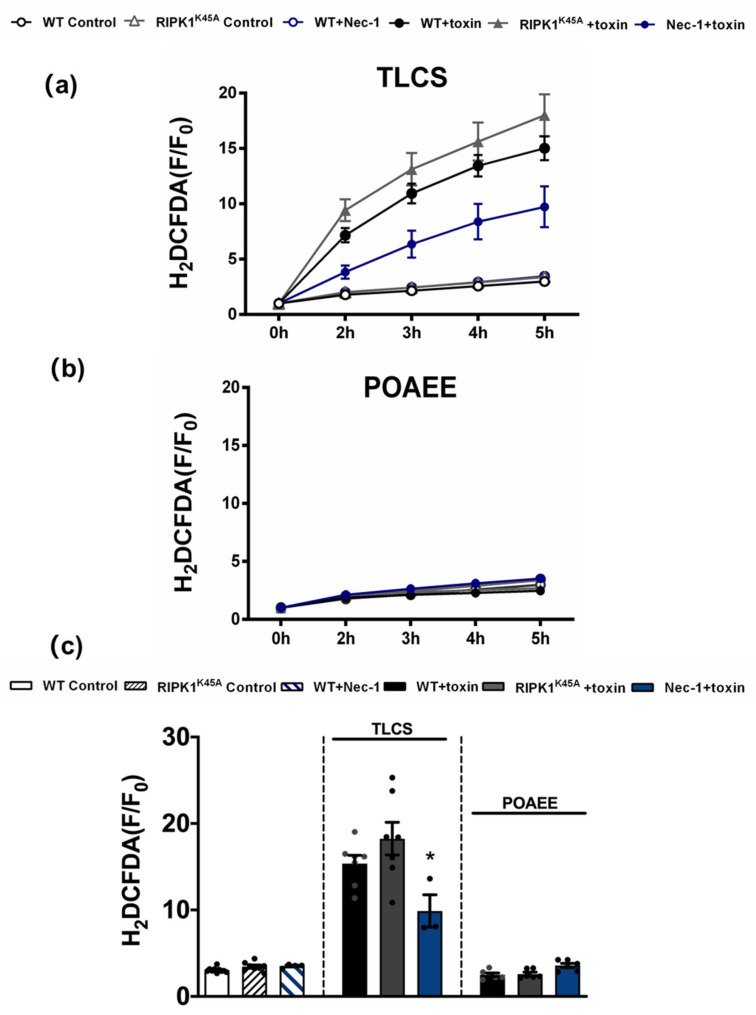
Inhibitory effects of Nec-1 on intracellular ROS in PACs. Line graphs showing the effects of RIPK1^K45A^ modification and Nec-1 (30 μM) on intracellular ROS levels (H_2_DCFDA) in the presence of (**a**) TLCS (500 μM) and (**b**) POAEE (100 μM). (**c**) Bar graph showing the endpoint effects of RIPK1^K45A^ modification and Nec-1 on intracellular ROS levels at 5 h in the presence of TLCS and POAEE. Each dot represents a mouse. Changes are normalized increases in fluorescence from the baseline (F/F_0_) and data expressed as the mean ± SEM (*n* ≥ 3 mice/group; n.b. some traces have overlap and the symbols are masked). Significant differences in Nec-1 from WT controls are shown as * *p* < 0.05.

Since TLCS has been shown to cause Ca^2+^-dependent ROS generation in both human and murine PACs [23], the effects of RIPK1 activity on intracellular ROS were assessed using the RIPK1^K45A^ modification and pharmacological inhibition (Figure 6). Basal ROS levels were similar in RIPK1^K45A^ and WT PACs. Application of 500 µM TLCS induced a ROS elevation in WT PACs; a trend to greater TLCS-induced ROS was observed in RIPK1^K45A^ PACs compared to WT, although this did not attain statistical significance. While Nec-1 (30 µM) did not affect basal ROS generation *per se*, it significantly decreased TLCS-induced ROS production. In contrast, application of POAEE (100 µM) did not increase ROS levels in either WT or RIPK1^K45A^ PACs, and 30 µM Nec-1 did not alter ROS levels in the presence of POAEE (Figure 6).

## 4. Discussion

Our results show that Nec-1 was highly effective in ameliorating pathological events in multiple AP models that reflect the principal aetiologies, including for the first time a protective action in alcoholic AP. Alcohol excess and gallstones together account for approximately 80% of clinical AP cases, however, no specific therapy is currently available to combat this debilitating and sometimes fatal disease. Previously Nec-1 has been evaluated as potential treatment for a range of pathologies, including ischemia-reperfusion injury [24,25,26,27,28], neurodegeneration [29,30], inflammatory disease [31,32], hepatitis [33] and lethal irradiation [34]. However, prior studies in AP assessing the action of Nec-1 and involvement of necroptosis have generated mixed findings. For example, while Nec-1 improved CER-AP, TLCS-AP [10] and L-arginine-induced AP [16], it was ineffective in two evaluations of CER-AP [15,35]. Such discrepancies may reflect differences in methodologies and/or variability in the delivery of Nec-1. In the present study, in order to ensure a stable and consistent Nec-1 application due to its relatively short half-life [36], the RIPK1 inhibitor was administered via osmotic mini-pump allowing an accurate comparison across three distinct AP models, as previously employed to investigate Orai1 inhibitors [22]. Using this standardised approach, our data demonstrate that Nec-1 is effective in multiple AP models when applied as a treatment i.e., after AP induction, strongly supporting further investigation of its actions to assist drug discovery.

RIPK1 is one of 28 kinases implicated in AP based on an interactions network derived from Gene Ontology Annotations [37]. Investigation of RIPK1 involvement by complete knockout of the protein is not feasible since these animals die shortly after birth [38]. Our results demonstrate that genetic modification of RIPK1 to a kinase dead variant (RIPK1^K45A^) did not mirror those of pharmacological inhibition, and indicate only a partial involvement of RIPK1-dependent necroptosis in AP. In accord, prior evaluations using genetic mutations showed that no protection was afforded by RIPK1^KD/KD D138N^ or by RIPK1 P-loop deficient RIP1^Δ/Δ^ mice in CER-AP [12,14]. In contrast, genetic deletion of RIPK3 or MLKL ameliorated both CER-AP and TLCS-AP [10,11,12,13]. Interestingly, evidence suggests that both RIPK1 and RIPK3 may not always be prerequisites for necroptosis to occur [39]. For example, TLR3-induced necroptosis in fibroblasts and endothelial cells did not require RIPK1 but involved both RIPK3 and MLKL [40], whilst necroptosis due to cytomegalovirus infection was RIPK1-independent but involved RIPK3 activity [41]. More recently, pancreatic tumour-promoting effects of RIPK1 were shown to be independent of its co-association with RIPK3 [42].

While recent evidence supports an involvement of necroptosis in the pathogenesis of AP, the contribution of RIPK1 appears variable and dependent on aetiology. Here the extent of RIPK1-dependent necroptosis differed according to the AP model, with pancreatic injury in RIPK1^K45A^ mice significantly reduced compared to WT in CER-AP. This contrasted with little or no protection observed in TLCS-AP and FAEE-AP, implying a more extensive participation of RIPK1 in CER-AP. In isolated PACs, the involvement of RIPK1 in cell death modalities was complex; whilst TLCS-induced PAC necrosis was reduced by the RIPK1^K45A^ modification, apoptosis was potentiated. The latter was linked to earlier release of cytochrome C from mitochondria in RIPK1^K45A^ mice, accompanied by ROS elevation, consistent with previous studies demonstrating ROS driven apoptosis in this cell type [23,43]. Previously deletion of X-linked inhibitor of apoptosis protein was shown to increase both RIPK1 degradation and caspase activity, resulting in enhanced PAC apoptosis and reduced necrosis that ameliorated AP [44]. Our findings of increased TLCS-induced apoptosis, with concurrent reduction of necrosis in RIPK1^K45A^ mice, are consistent with an alteration of the apoptosis-necrosis balance that influences AP severity [23,45,46,47].

In contrast to genetic manipulation, Nec-1 application reduced both apoptotic and necrotic PAC death, indicating multiple actions that determine responses to pathophysiological stimulation. Previously, TLCS-induced necrosome formation, ATP depletion and cell death in PACs were reduced by Ca^2+^ chelation with BAPTA pre-treatment [10]. In the present study Nec-1 did not alter the sustained [Ca^2+^]_c_ elevations caused by TLCS in PACs, implying protective actions distal to Ca^2+^ release and store-operated Ca^2+^entry mechanisms engaged by bile acids [48,49]. Interestingly, the Ca^2+^-dependent ROS elevations induced by TLCS [23] were reduced by Nec-1, consistent with reported inhibitory actions on acetaminophen-induced ROS production and mitochondrial dysfunction in hepatocytes [50], and TNFα-induced ROS accumulation and cell death in NF-κB activation-deficient cells [51]. The ability of Nec-1 to decrease TLCS-induced ROS is likely to be of importance to its beneficial actions in AP. A recent study has demonstrated a role of transient receptor potential melastatin 2 (TRPM2) channels to detrimental actions of bile acids on acinar cells that are mediated through ROS elevation [52].

The effects of Nec-1 application in disease studies have largely been attributed to RIPK1 inhibition. Our present findings strongly indicate that multiple mechanisms contribute to its protective effects in AP; to-date no investigations have addressed the possible involvement of collateral targets of Nec-1 in AP. Amongst those inhibited by Nec-1 is IDO, the initial, rate-limiting component of the kynurenine pathway involved in cellular tryptophan metabolism, known to have a complex role in inflammation and disease [53,54]. Early studies showed that inhibition of IDO could increase the severity of diseases, including asthma [55], uveoretinitis [56] and colitis [57]. However, investigations have also demonstrated that IDO1 deficiency/inhibition reduced inflammation and metaplasia in chronic gastric inflammation [58], the development of allergic airway disease [59], and ameliorated rheumatoid arthritis symptoms via a diminished autoreactive B cell response [60]. In the present study, treatment with EPA, a highly selective and potent IDO1 inhibitor [61], was protective against pancreatic damage in TLCS-AP, indicating that actions on this enzyme likely contribute to beneficial effects of Nec-1. Although the expression of IDO was not evaluated here, upregulation of both IDO1 and IDO2 have been shown in human pancreatic ductal adenocarcinoma [62,63] and a more detailed evaluation of IDO involvement in AP is the focus of a separate, on-going study. The present findings are consistent with recent studies which have demonstrated the importance of the kynurenine pathway to AP. Increased plasma levels of 3-hydroxykynurenine in AP patients paralleled disease severity in clinical AP [64], while inhibition of kynurenine-3-monooxygenase, an enzyme downstream of IDO, prevented multiple organ failure in rodent AP models [18]; a series of kynurenine-3-monooxygenase inhibitors is now in development for AP therapy [17]. Interestingly, it has also been shown that Nec-1 exerts cyclophilin D-dependent beneficial actions in cardiac ischaemia-reperfusion injury [65]. A collateral inhibitory effect of Nec-1 on cyclophilin D would also be advantageous in AP, since genetic deletion (*ppif^−/−^*) and pharmacological inhibition of this protein prevented mitochondrial permeability transition pore (MPTP) formation in the exocrine pancreas and protected against AP [3,66,67,68,69]. Thus, multiple actions of Nec-1 provide an aggregation of complementary beneficial effects of potential therapeutic value in AP and serve as a starting point for development of novel agents.

In conclusion, our study has demonstrated important protective actions of Nec-1 in multiple AP models, including for the first time in alcoholic AP. However, RIPK1 kinase modification only partially reflected the effects of Nec-1. The efficacy of Nec-1 treatment indicates that inhibition of multiple targets might be an effective therapeutic strategy in AP and further investigation is warranted.

## Data Availability

The data presented in this study are available on request from the corresponding author.

## Supplementary Materials

The following are available online at https://www.mdpi.com/article/10.3390/cells10051035/s1, Figure S1: Expression and distribution of RIPK1 and RIPK3 in RIPK1K45A and strain-matched WT PACs.
