# Supplementary material for: Protective Effects of Necrostatin-1 in Acute Pancreatitis: Partial Involvement of Receptor Interacting Protein Kinase 1
Source: Cells. 2021 Apr 27;10(5):1035. doi: 10.3390/cells10051035 (PMC8145347; doi:10.3390/cells10051035)
Supplement: Supplementary file 1 [file cells-10-01035-s001.zip › cells-1156529-supplementary.pdf]

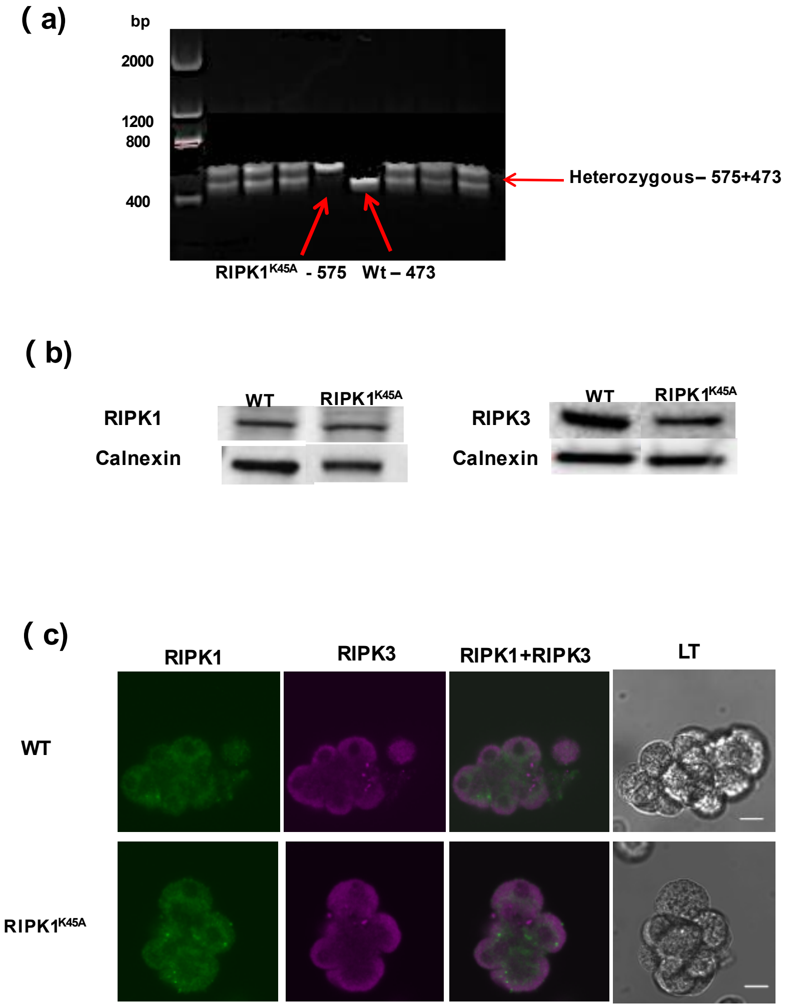

Figure S1. Expression and distribution of RIPK1 and RIPK3 in RIPK1K45A and strain-matched WT PACs. (**a**) PCR experiments distinguished WT, heterozygous and homozygous RIPK1K45A mice. Each lane represents a single mouse (575 bp: RIPK1K45A homozygous; 473 bp: WT and both 575 bp and 473 bp: RIPK1K45A heterozygous). (**b**) Representative western blotting images of RIPK1 (60 KD) and RIPK3 (57 KD) in WT and RIPK1K45A PACs. Calnexin (92 KD) was used as a loading control. (**c**) Immunofluorescence images of RIPK1 (green) and RIPK3 (magenta) showing distribution in isolated PACs from WT and RIPK1K45A (scale bar: 10 μm). All data are representative of at least 3 experiments.
